# Review on plant antimicrobials: a mechanistic viewpoint

**DOI:** 10.1186/s13756-019-0559-6

**Published:** 2019-07-16

**Authors:** Bahman Khameneh, Milad Iranshahy, Vahid Soheili, Bibi Sedigheh Fazly Bazzaz

**Affiliations:** 1 Department of Pharmaceutical Control, School of Pharmacy, Mashhad University of Medical Sciences, Mashhad, Iran; 2 Department of Pharmacognosy, School of Pharmacy, Mashhad University of Medical Sciences, Mashhad, Iran; 3 Biotechnology Research Center, Pharmaceutical Technology Institute, Mashhad University of Medical Sciences, Mashhad, Iran

**Keywords:** Antibiotic-resistant, Antimicrobial activity, Combination therapy, Mechanism of action, Natural products, Phytochemicals

## Abstract

Microbial resistance to classical antibiotics and its rapid progression have raised serious concern in the treatment of infectious diseases. Recently, many studies have been directed towards finding promising solutions to overcome these problems. Phytochemicals have exerted potential antibacterial activities against sensitive and resistant pathogens via different mechanisms of action. In this review, we have summarized the main antibiotic resistance mechanisms of bacteria and also discussed how phytochemicals belonging to different chemical classes could reverse the antibiotic resistance. Next to containing direct antimicrobial activities, some of them have exerted in vitro synergistic effects when being combined with conventional antibiotics. Considering these facts, it could be stated that phytochemicals represent a valuable source of bioactive compounds with potent antimicrobial activities.

## Introduction

Today’s, microbial infections, resistance to antibiotic drugs, have been the biggest challenges, which threaten the health of societies. Microbial infections are responsible for millions of deaths every year worldwide. In 2013, 9.2 million deaths have been reported because of infections i.e. about 17% of total deaths [1, 2]. The occurrence of the evolution of resistance has caused the existing antibacterial drugs to become less effective or even ineffective [3, 4]. In recent years, various strategies have been suggested to overcome the resistance of antibiotics. One of the recommended strategies to achieve this goal has involved the combination of other molecules with the failing antibiotics, which apparently restores the desirable antibacterial activity [5, 6]. These molecules can be non-antibiotic drugs with potential antibacterial properties that can create opportunities for innovative therapeutic approaches [7]. In regards to this case, phytochemicals have exhibited potent activities while many researchers have used natural products to act against bacterial resistance [8–11]. These agents can act alone or in combination with antibiotics to enhance the antibacterial activity against a wide range of bacteria [10, 12–14]. However, up to this date, the structure-activity relationships and mechanisms of action of natural compounds have largely remained elusive. In the present review, we have focused on describing the relationship between the structure of natural compounds and their possible mechanism of action.

### Mechanisms of antibacterial activity and resistance

**Fig. 1:**
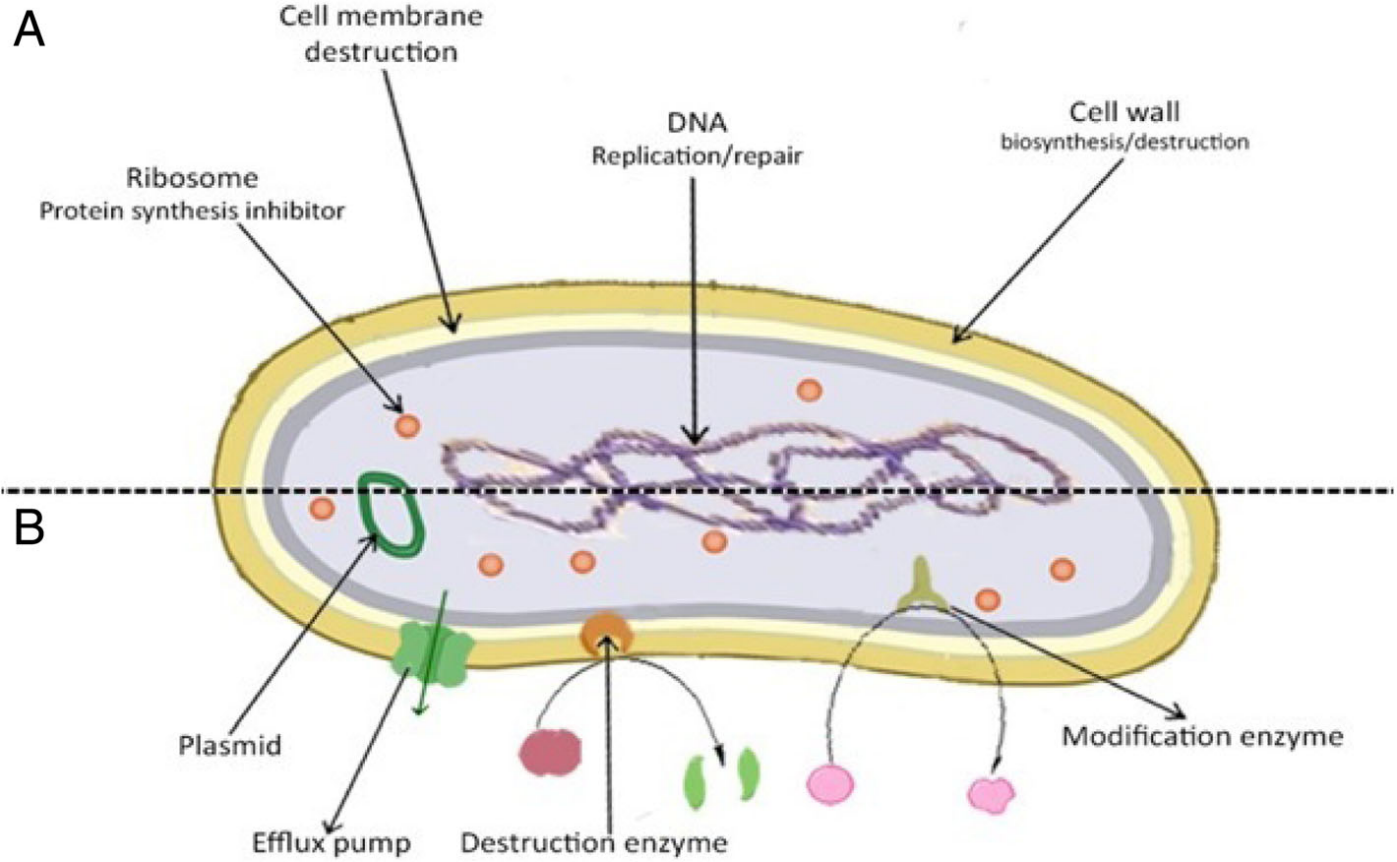
**a** Proven targets for antibacterial drugs. Protein biosynthesis at the ribosome is targeted by different classes of antibiotics such as macrolides, tetracyclines, aminoglycosides. Cell membrane can be targeted by some antibiotics such as Polymyxin B. These antibiotics alter bacterial outer membrane permeability and finally destabilize outer membrane of bacteria. The fluoroquinolone antibiotics inhibit DNA replication by trapping a complex of DNA bound to the enzyme DNA Gyrase. Cell-wall biosynthesis is inhibited by the various classes of antibiotics. **b** Multiple antibiotic resistance mechanisms in bacteria. Efflux pumps remove the antibiotics from bacteria (e.g. Fluoroquinolones and trimethoprim resistance in *P. aeruginosa*). Destruction enzymes that degrade the antibiotics (β-lactams in *Enterobacteriaceae*). Modifying enzymes which change the antibiotic structure (e.g. chloramphenicol or fosfomycin in *P. aeruginosa*)

The antibacterial activity of an agent is mainly attributed to two mechanisms, which include interfering chemically with the synthesis or function of vital components of bacteria, and/or circumventing the conventional mechanisms of antibacterial resistance. Figure 1 shows these mechanisms and as it can be observed, there are multiple targets for the antibacterial agents that comprise (Ι) bacterial protein biosynthesis; (ΙΙ) bacterial cell-wall biosynthesis; (ΙΙΙ) bacterial cell membrane destruction; (ΙV) bacterial DNA replication and repair, and (V) inhibition of a metabolic pathway.

In addition, bacteria may show resistance to antibacterial agents through a variety of mechanisms. Some bacterial species are innately resistant to one or more classes of antimicrobial agents. In these cases, all strains of that bacterial species exhibit resistant to all the members of those antibacterial classes. A major concern is that the bacteria acquire resistance, where initially susceptible bacterial populations become resistant to the antibacterial agent [15]. So that, one of the key factors in finding the solutions for slowing the development of antibiotic-resistant is knowing about the mechanisms of antibacterial resistance [16], which mainly include the activation of efflux pump, destroying the antibacterial agents through the destruction enzymes, modification of antibiotics by the means of modifying enzymes, and the alteration of target structures in the bacterium which have lower affinity for antibacterial recognition [8]. It should be also noted that the resistance to antibacterial agents can be related to one kind of mechanism or different types together. The main mechanism for spreading the resistance of antibiotic through bacterial populations is plasmids in the role of genetic material, which are capable of being independently replicated and passed between bacterial cells and species.

Each of these mechanisms has been separately discussed in the following.

### Mechanisms of action of antibacterial agents

#### Bacterial protein biosynthesis

There is a vast number of molecular steps involved in initiation, elongation, and termination of protein assembly by the bacterial ribosome. Therefore, the inhibition of protein synthesis by targeting the ribosomal subunits is an effective approach to combat bacterial infections. Important classes of antibiotics such as macrolides, tetracyclines, aminoglycosides, and oxazolidinones show antibacterial activities through this particular mechanism [17].

#### Cell-wall biosynthesis

The bacterial cell wall layer stands as a proven target for antibacterial agents, which consists a network of peptide and glycan strands that are covalently cross-linked to each other and can provide higher mechanical strength for osmotic lysis. There are two types of family enzymes that have critical roles in the formation of this layer, which include transglycosylases and transpeptidases while their functionality has been previously described. Bifunctional enzymes that contain both domains of transpeptidase and transglycosylase are suitable targets for bactericidal antibiotics including penicillins and cephalosporins. The family of Glycopeptide antibiotic, such as vancomycin, has been also observed to target the peptidoglycan layer within cell-wall assembly through another way. These antibiotics are able to tie up the peptide substrate of peptidoglycan layer and thus, prevent the occurrence of a reaction with enzymes. However, the net effect is quite similar, which reduces the cross-linking of peptidoglycan and consequently weaken the cell wall [18].

Filamenting temperature-sensitive mutant Z (FtsZ) is the first protein to move towards the division site during the process of cell division. This protein is necessary for recruiting other proteins that ultimately produce a new cell wall between the dividing bacterial cells [19]. So far, one of the promising approaches for the purpose of combating bacterial infections has been the procedure of targeting at the inhibition of bacterial cell division, which is carried out by controlling the FtsZ functionality.

#### Inhibiting nucleic acid synthesis

DNA gyrase is known as the enzyme that is responsible for performing the supercoiling and uncoiling of bacterial DNA and DNA replication. This enzyme is essential for synthesis, replication, repair, and transcription procedures and consequently, gyrase can be considered as a fine target for antibacterial agents and antibiotics including nalidixic acid, as well as fluoroquinolones such as ciprofloxacin [20].

#### Destruction of bacterial membrane

Various antibiotics, such as polymyxins, can bind to the lipid A component of lipopolysaccharide and therefore, cause structural alterations by the means of phospholipid exchange that would result in an osmotic imbalance and finally rapid bacterial death [15].

Bacterial cell membrane destruction has been reported from a long time ago, which had involved even other chemical compounds such as local anesthetics [21] or disinfectants [22]. Destruction of external membrane, cytoplasmic membrane, and energy metabolism of cells can cause the loss of permeability, leakage of intracellular constituents and even the coagulation of cytoplasm.

### Mechanisms of resistance to antibacterial agents

#### Efflux pump

An antibacterial agent can be effective upon reaching the specific site of action and accumulate at specific concentrations. Efflux pumps (EPs) act as an export or efflux system that can cause resistance to the wide ranges of antibacterial agents. Throughout this mechanism, the antibacterial agent is pumped out faster than the time it requires to be diffused in bacterial cell and consequently, the intrabacterial concentration becomes much less than the effective value. For example, the protein-synthesis systems such as ribosome are located in the cytoplasm. So that, inhibitors of protein synthesis are forced to pass through the cell membranes and then accumulate up to a sufficient concentration to induce the blockade of protein synthesis. By reducing the intrabacterial concentration of inhibitors, which are mediated by EPs, the procedures of bacterial protein synthesis can be performed without any interruptions [23, 24].

EPs are capable of conveying both lipophilic or amphipathic molecules out of the bacteria. In another aspect, they have been also able to transport one type of substrate and/or the range of structurally dissimilar antibacterial agents (e.g. multiple classes of antibiotics), which had been detected and found in multiple drug-resistant bacteria [25].

**Fig. 2:**
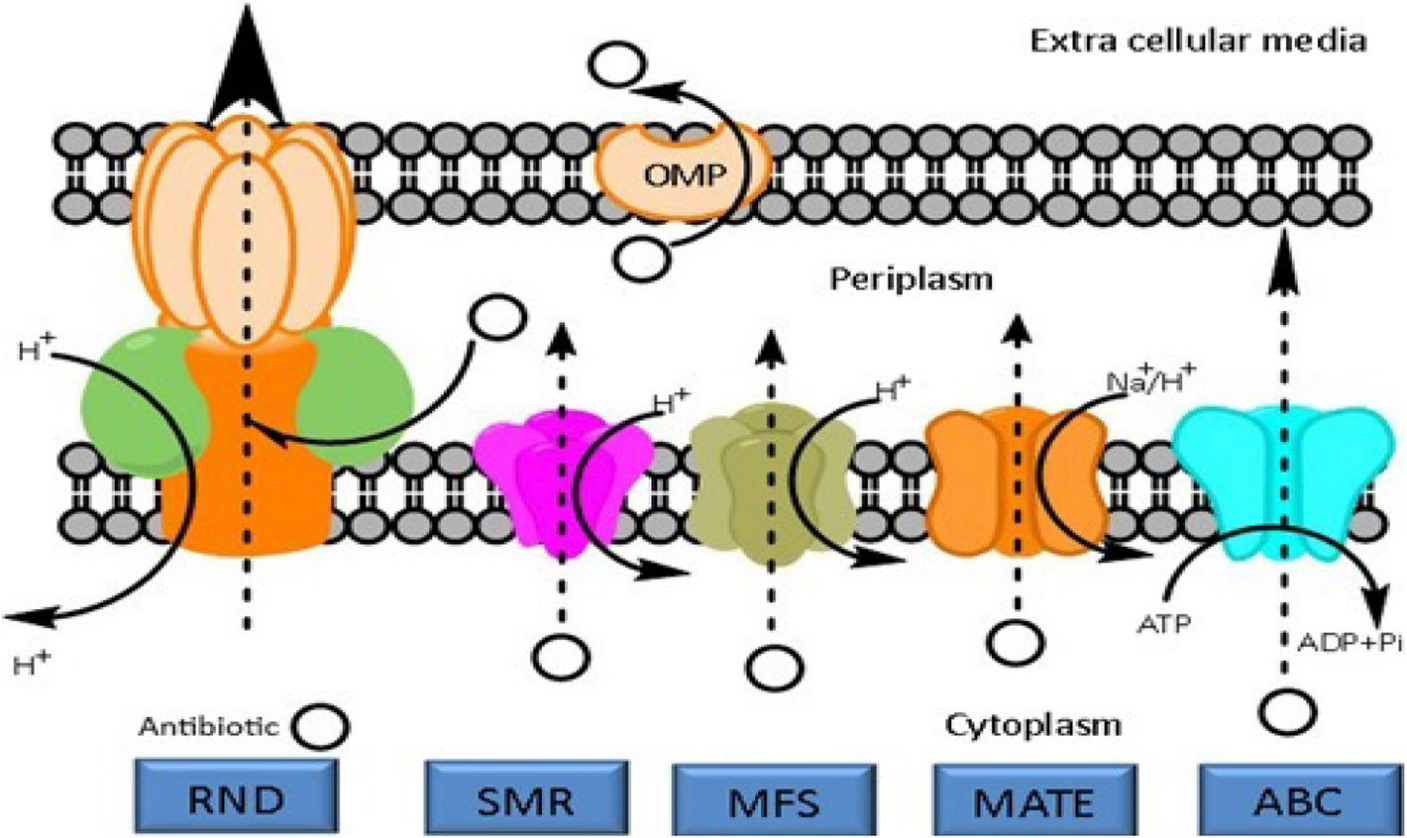
Schematic representation of the main families of bacterial efflux pumps. The resistance- nodulation-division (RND) family, the small multidrug resistance (SMR) family, the major facilitator superfamily (MFS), the multidrug and toxic compound extrusion (MATE) family and the adenosine triphosphate (ATP)-binding cassette (ABC) superfamily

Five major families of EPs have been recognized in bacteria, which includes major facilitator superfamily (MFS), multidrug and toxic efflux (MATE), resistance-nodulation-division (RND), small multidrug resistance (SMR), and ATP binding cassette (ABC) [26]. The MFS, ABC, SMR, and MATE families are mainly found in both Gram-positive and -negative bacteria, while the RND superfamily is specifically found in Gram-negative bacteria [27]. The group of RND families always consists of a tripartite complex that spans across both membranes of Gram-negative bacteria. In regards to Gram-positive bacteria, the MFS family has been reported as the most abundant EPs while their well-known members are known to be NorA from *Staphylococcus aureus* and PmrA from *Streptococcus pneumoniae*. Figure 2 shows the mentioned major families of EPs that exist within bacteria. Antibiotic resistance via this mechanism can be observed in a wide range of pathogenic Gram-positive and -negative bacteria and fungi such as *S. aureus*, *Pseudomonas aeruginosa*, *Acinetobacter baumannii*, and *Candida albicans* [28]. Therefore, employing EP inhibitors, EPIs, in combination with antibacterial agents can be contemplated as an effective approach for the purpose of combating microbial infections.

#### Structural modification of porins

Intracellular access of an antibiotic can be restricted by the reduction of antibiotics influx. Influx is mainly controlled by porins which are proteins able to form water-filled open channels that allowing the passive transportation of molecules across lipid bilayer membranes [29–31]. So, porins can be considered as potential targets for bactericidal compounds especially for Gram-negative bacteria [30]. Variation in porin structure results in alteration of membrane permeability and is a mechanism to escape from antibacterial agents [31]. This circumstance is one of the bacterial strategies for the antibacterial resistance which is frequently found in Gram-negative clinical pathogens such as *Acinetobacter* spp. and *Pseudomonas* spp. [29].

#### Destroying the antibacterial agents

The second strategy of bacterial resistance is the chemical degradation of antibiotics or antibacterial agents, in which unlike the previous one, the aim is to change the chemical formula. The classic degradation is mediated by attacking the hydrolytic enzyme, β-lactamase, to the β-lactam ring of penicillins, cephalosporins and carbapenems [32, 33]. In accordance with the observations, each enzyme molecule is able to hydrolyse 10^3^ antibiotic molecules per second; therefore, it can be stated that by the secretion of 10^5^ enzymes via resistant bacteria, 100 million molecules of the designated antibiotic are destroyed in every second and result in the complete ineffectiveness of the antibiotic [16].

#### Modification of antibiotics

Other classes of antibiotics, such as aminoglycosides, represent another mechanism of resistance with respect to the previous ones. These antibacterial agents are deactivated through the modification of functional groups at three sites by utilizing three kinds of modifying enzymatic. These modified products have displayed considerably lower affinity for RNA and have caused the blockage of protein synthesis since they are not capable of binding to ribosomes [34].

#### Altered target

Drug-binding site alteration can be counted as another resistance mechanism, in which the targeted site of antibacterial agent would be constructed in a form that the antibacterial agent is not able to react with it and thus, result in a dramatic reduction in the antibacterial activity of the agent. This type of resistance can be labeled as “Reprogramme the target structure” that is found in a wide range of resistant bacteria [16, 35]. To be stated as an example, resistance to erythromycin in resistant bacteria is attributed to different mechanisms such as the activation of efflux pump, as well as the alteration of drug action site that is known as peptidyl transferase. Throughout the methylation of this enzyme, which takes place at a specific amino acid residue, the procedures of protein biosynthesis that are mediated by antibiotics do not impair but the affinity of antibiotics to action site, peptidyl transferase, faces a remarkable reduction. This mechanism of resistance stands as the main resistance approach in drug-resistant clinical isolates of S. aureus [36].

Penicillin resistance can be occurred by expression of new forms of penicillin-binding proteins (PBPs), which contain a lower affinity for an antibiotic, via the mutation of corresponding genes. The acquisition of mecA gene in *S. aureus* species can lead to the production of new PBPs forms with low affinity for all of the β-lactam antibiotics. This type of mechanism has been widely observed in methicillin-resistant *Staphylococcus aureus* (MRSA) strains [37, 38].

An additional example of this mechanism is vancomycin resistance in Vancomycin-resistant enterococci (VRE) species. In these species, the vanHAX genes code a new pathway of enzymes which induce structural alterations by switching from the amide linkage in the D-Ala-D-Ala peptidoglycan structure to the ester linkage in the D-Ala-D-Lac structure resulting in reducing the drug-binding affinity up to 1000-fold [39].

### Plant-derived chemicals

Although synthetic antimicrobial agents have been already approved in many countries, yet the usage of natural compounds that are derived from microbial, animals, or plants attracts the attention of many researchers [40, 41]. These compounds have exhibited promising results in overcoming the emergence of antibiotic resistance in bacterial pathogens [42]. Among all of the available options, plant-derived compounds have displayed more potential applications in combating bacterial infections. Plant-derived chemicals are a wide group of chemical compounds that have been found naturally in plants. The extensive existence of these compounds has demonstrated beneficial advantages in terms of antioxidant, antibacterial, and antifungal activities. They can restore the clinical application of older antibiotics by increasing their potency and as a consequent, avoid the development of resistance [43]. Some of the plants and/or plant components which containing antimicrobial activities and are commercially available to consumers are listed in Table 1.

**Table 1:** Some of plant products with antimicrobial activity

Common name	Scientific name	Compound	Active against	Dosage form
Barberry	*Berberis vulgaris*	Berberine	Bacteria, protozoa	Soft gel 1000 mg
Black pepper	*Piper nigrum*	Piperine	Fungi, *Lactobacillus*, *Micrococcus*, *E. coli*, *E. faecalis*	
Burdock	*Arctium lappa*		Bacteria, fungi, viruses	Capsule 475 mg
Caraway	*Carum carvi*		Bacteria, fungi, viruses	Capsule 1000 mg
Cascara sagrada	*Rhamnus purshiana*	Tannins	Bacteria, fungi, viruses	Capsule 425, 450 mg
Chamomile	*Matricaria chamomilla*	Anthemic acid	*M. tuberculosis*, *S. typhimurium*, *S. aureus*	
Clove	*Syzygium aromaticum*	Eugenol	General	Capsule 500 mg
Cranberry	*Vaccinium* spp.	Fructose	Bacteria	Capsule 500 mg
Eucalyptus	*Eucalyptus globulus*	Tannin	Bacteria, viruses	Inhaler and tablet
Garlic	*Allium sativum*	Allicin, ajoene	General	Tablet
Goldenseal	*Hydrastis canadensis*	Berberine, hydrastine	Bacteria, *Giardia duodenale,* Trypanosomes	Solution, 500 mg per dosage
Green tea	*Camellia sinensis*	Catechin	General	
Licorice	*Glycyrrhiza glabra*	Glabrol	*S. aureus*, *M. tuberculosis*	Capsule 450 mg
Oak	*Quercus rubra* *Allium cepa*	Tannins Quercetin		Capsule 500, 650 mg
Onion	*Allium cepa*	Allicin	Bacteria, *Candida*	
Oregon grape	*Mahonia aquifolia*	Berberine	*Plasmodium* Trypansomes, general	Capsule 500 mg
Senna St. John’s wort	*Hypericum perforatum*	Hypericin, others	General	Table 450 mg
Thyme	*Thymus vulgaris*	Caffeic acid Thymol Tannins	Viruses, bacteria, fungi	Capsule 450 mg
Turmeric	*Curcuma longa*	Curcumin, Turmeric oi	Bacteria, protozoa	

Based on their chemical structures, they can be classified into several major groups that include alkaloids, sulfur-containing compounds, terpenoids, and polyphenols. The most important phytocompounds from different chemical classes have been listed in Table 2.

**Table 2:** The strongest plant antimicrobial compounds reported in recent years

Class of naturally compound	Compound	Conc.	Mechanisms of action	Active against	Findings	Reference
Alkaloids	Reserpine	100 mg/L	Efflux pump inhibitor	*Staphylococcus sp.*, *Streptococcus sp.* and *Micrococcus sp.*	• Reducing the (MIC) of antibiotics • *In vitro* model	[60]
	Piperine	100μg/mL	Efflux pump inhibitor	Methicillin resistant *Staphylococcus aureus* (MRSA) and *Staphylococcus aureus*	• *In vitro* model	[46]
	Berberine	4 mM	Cell division inhibitor, Protein and DNA synthesis inhibitor	*Escherichia coli* *Candida albicans*	• *In vitro* model	[51, 196]
	Chanoclavine		Efflux pump inhibitor	*E. coli*	• Reducing the MIC of tetracycline up to 16-folds	[70]
	Solasodine	32μg/mL	Destruction of bacterial membrane	*C. albicans*	• Potent fungicidal activity • *In vitro* model	[197]
	Conessine	20 mg/L	Efflux pump inhibitor	*Pseudomonas aeruginosa*	• Active against RND family pump	[71]
	Evocarpine	5 mg/mL		*Mycobacterium tuberculosis*		[198]
	Tomatidine		ATP synthase inhibitor	*Listeria, Bacillus and Staphylococcus spp*	• *In vitro* model • Its analog possess bactericidal activities	[199, 200]
	Lysergol		Efflux pump inhibitor	*E. coli*	• Lowering the dose of antibiotics	[201]
Organosulfur	Allicin		Sulfhydryl-dependent enzyme inhibitor, DNA and protein synthesis inhibitor	*Staphylococcus epidermidis,* *P. aeruginosa,* *Streptococcus agalactiae*	• In the gas phase active against antibiotic resistant strains	[83]
	Ajoene		Sulfhydryl-dependent enzyme inhibitor	*Campylobacter jejuni, Streproproteus, Staphylococcus* and *E. coli*	• Zone inhibition method • More potent than allicin	[88]
	Sulforaphane		Destruction of bacterial membrane, ATP synthase inhibitor, DNA and protein synthesis inhibitor	*E. coli*	• Did not destroy the membrane integrity directly	[202]
	Berteroin	Range of 1 – 16 μg/mL		*Helicobacter pylori*		[108]
	Hirsutin	Range of 8 – 16 μg/mL		*P. aeruginosa* and *Bacillus cereus*	• Having antifungal and antimicrobial activities.	[203]
	Alyssin			*H. pylori*		[108]
	Erysolin	Range of 4 – 32 μg/mL		*H. pylori*		[108]
	Allyl isothiocyanate, Benzyl isothiocyanate and Phenethyl isothiocyanate			*Bacillus subtilis, S. aureus, S. epidermidis, Enterococcus faecalis, Salmonella typhimurium, Enterobacter aerogenes, Enterobacter cloacae, and E. coli*	• Show antibacterial activity against foodborne and resistant pathogens. • AITC was the major ITC in the stem and leaf of *R. sativus*	[204]
Phenolic compounds	Resveratrol	0.064, 0.313 mg/mL	Efflux pump inhibitor	*Mycobacterium smegmatis, Campylobacter jejuni*	• Reduced MIC value of antibacterial agent against resistant strain	[112-113]
	Baicalein	64, 128, 64 μg/mL	Efflux pump inhibitor	*M. smegmatis,* MRSA*, C. albicans*	• Reduced MIC value of antibacterial agent against resistant strain	[113, 117, 205]
	Biochanin A	256 μg/mL, no inhibitory effect, 12 μM	Efflux pump inhibitor	*M. smegmatis*, MRSA, Chlamydia spp.	• Reduced MIC value of antibacterial agent against resistant strain	[113, 119-120]
	Formononetin	256 μg/mL	Efflux pump inhibitor	*M. smegmatis*	• Reduced MIC value of antibacterial agent against resistant strain	[113]
	Luteolin	32 μg/mL	Efflux pump inhibitor	*Mycobacteria spp.*	• Reduced MIC value of antibacterial agent against resistant strain	[113, 206]
	Kaempferol	125, 128-256 μg/mL	Efflux pump inhibitor	MRSA*, C. albicans,*	• Reduced MIC value of antibacterial agent against resistant strain	[126-127]
			Rigidifing bacterial membrane	*E. coli*		[139]
	Kaempferol rhamnoside	1.56 μg/mL	Efflux pump inhibitor	*S. aureus*	• Increased antimicrobial activity of ciprofloxacin	[128]
	Myricetin	32 μg/mL	Efflux pump inhibitor	*M. smegmatis*		[113]
	Rhamentin	19-75 μg/mL	Efflux pump inhibitor	*S. aureus*		[129]
	Quercetin	75 μg/mL	Efflux pump inhibitor	*S. aureus*		[129]
		48.5 and 19.9μM	d-Alanine:d-alanine ligase	*H. pylori* and *E. coli*		[144]
	Chrysosplenol-D	25 μg/mL	Efflux pump inhibitor	*S. aureus*	• Inhibited NorA EP in the presence of subinhibitory concentrations of berberine	[122]
	Chrysoplentin	6.25 μg/mL	Efflux pump inhibitor	*S. aureus*	• Inhibited NorA EP in the presence of subinhibitory concentrations of berberine	[122]
	Silybin		Efflux pump inhibitor	*S. aureus*		[124]
	Biochanin A	10 μg/mL	Efflux pump inhibitor	*S. aureus*	• Reduced the expression of NorA protein	[123]
	Genistein	10 μg/mL	Efflux pump inhibitor	*S. aureus*		
	Orobol	10 μg/mL	Efflux pump inhibitor	*S. aureus*		
	4′,6′-Dihydroxy-3′,5′-dimethyl-2′-methoxychalcone	10 μg/mL	Efflux pump inhibitor	*S. aureus*	• Reduced MIC of erythromycin from 0.4 to 0.1 μg/mL	[130]
	4-phenoxy-4′-dimethylamino ethoxychalcone	9 μM	Efflux pump inhibitor	*S. aureus*	• Equipotent to reserpine	[131]
	4-dimethylamino-4′-dimethylamino ethoxychalcone	7.7 μM	Efflux pump inhibitor	*S. aureus*	• Equipotent to reserpine	[131]
	Bergamottin epoxide	35.7 μg/mL	Efflux pump inhibitor	MRSA	• Resulted in the 20-fold reduction in MIC value of norfloxacin	[168]
	5,7-dihydroxy-6-(2-methylbutanoyl)-8-(3-methylbut-2-enyl)-4-phenyl-2*H*-chromen-2-one	8 μg/mL	Efflux pump inhibitor	MRSA		[168]
	5,7-dihydroxy-8-(2-methylbutanoyl)-6-(3-methylbut-2-enyl)-4-phenyl-2*H*-chromen-2-one	8 μg/mL	Efflux pump inhibitor	MRSA		
	Epigallocatechin gallate	1-10 μM	DNA gyrase	-		[134]
		200 μM	Beta-ketoacyl-[acyl carrier protein] reductase (FabG)	*E. coli*		[141]
		64 μg/mL	Inhibition of dihydrofolate reductase	*Stenotrophomonas maltophilia*		[148]
	Chebulinic acid		DNA gyrase	*M. tuberculosis*	*In silico*	[135]
	3-p-Trans-coumaroyl-2-hydroxyquinic acid	2.5-10 mg/mL	Damage to the cytoplasmic membrane	*S. aureus*	• Active against eleven food-borne pathogens	[137]
	p-Coumaric acid		Damage to the cytoplasmic membrane	*Oenococcus oeni* and *Lactobacillus hilgardii*		[138]
	Apigenin	132.7 and 163.0 μM	d-Alanine:d-alanine ligase	*H. pylori* and *E. coli*	• Reverse inhibitor and competitive with ATP	[144]
	Sophoraflavanone B	15.6-31.25 μg/mL	Direct interaction with peptidoglycan	MRSA	-	[145]
	Naringenin	256 μg/mL	Beta-Ketoacyl acyl carrier protein synthase (KAS) III	*E. faecalis*	• Showed activity against vancomycin resistance *E. faecalis*	[140]
	Eriodictyol	256 μg/mL	Beta-Ketoacyl acyl carrier protein synthase (KAS) III	*E. faecalis*	• Showed activity against vancomycin resistance *E. faecalis*	[140]
	Taxifolin	128 μg/mL	Beta-Ketoacyl acyl carrier protein synthase (KAS) III	*E. faecalis*	• Showed activity against vancomycin resistance *E. faecalis*	[140]
	Sakuranetin	2.2 μM	FabZ	*H. pylori*		[142]
	3,6-Dihydroxyflavone	16-32 μM	Beta-Ketoacyl acyl carrier protein synthase (KAS) III and I	*E. coli*	• High binding affinity with KAS III	[109]
	Curcumin	13.8 μg/mL	Sortase A	*S. aureus*	• No growth inhibitory activity	[149]
		25-100 μM	leaky membrane	*S. aureus* and *E. coli*	• Broad spectrum activity	[143]
	Morin	39.37 and 8.54 μM	Sortase A and B	*S. aureus*	• Reduced clumping activity	[150]
	4′,7,8-trihydroxyl-2-isoflavene	0.85 μM	urease inhibitor	*H. pylori*	• 20-fold lower than acetohydroxamic acid	[147]
Coumarin	Aegelinol	16 μg/mL	DNA gyrase inhibitor	*Salmonella enterica serovar Typhi, Enterobacter aerogenes, Enterobacter cloacae, S. aureus*	• Higher activity against Gram-negative bacteria than Gram-positive ones particularly *Salmonella thypii*	[157]
		Dose dependent inhibition between 5 and 25 μg/mL		*H. pylori*		
	Agasyllin	32 μg/mL	DNA gyrase inhibitor	*S. enterica serovar Typhi, E. aerogenes, E. cloacae, S. aureus*	• Higher activity against Gram-negative bacteria than Gram-positive ones particularly *Salmonella thypii*	[157]
		Dose dependent inhibition between 5 and 25 μg/mL		*H. pylori*		
	4′-senecioiloxyosthol	5 μg/mL	DNA gyrase inhibitor	*B. subtilis*	• 6-fold more active against *B. subtilis* ATCC 9372 than that of xanthotoxin	[158]
	Osthole	125 μg/mL	DNA gyrase inhibitor	*B. subtilis, S. aureus, K. pneumoniae,* MSSA	-	[158]
	Asphodelin A 4′-O-β-D-glucoside	Range of 128–1024 μg/mL	DNA gyrase inhibitor	*S. aureus, E. coli, P. aeruginosa, C. albicans, Botrytis cinerea*	-	[160]
	Asphodelin A	Range of 4–128 μg/mL	DNA gyrase inhibitor	*S. aureus, E. coli, P. aeruginosa, C. albicans, B. cinerea*	-	[160]
	Clorobiocin	-	DNA gyrase inhibitor	-	• noviosyl sugar moiety is essential for biological activity • mutations at Arg136 of GyrB in *E. coli* results in coumarin-resistant	[161]
	Novobiocin	-	DNA gyrase inhibitor	-	• noviosyl sugar moiety is essential for biological activity • mutations at Arg136 of GyrB in *E. coli* results in coumarin-resistant	[161]
	Coumermycin A1	-	DNA gyrase inhibitor	-	• noviosyl sugar moiety is essential for biological activity • mutations at Arg136 of GyrB in *E. coli* results in coumarin-resistant	[161]
	Bergamottin epoxide	-	Efflux pump inhibitor	MSRA	• 20-fold reduction in the MIC value of norfloxacin against MRSA	[169]
	6-Geranyl coumarin	No inhibitory effect	Efflux pump inhibitor	*S. aureus*	• Reduced the MIC for tetracycline and norfloxacin by 2 times	[170]
	Galbanic acid	No inhibitory effect	Efflux pump inhibitor	MDR clinical isolates of *S. aureus*	• Reduced MIC range of ciprofloxacin and tetracycline from 10-80 μg/ml to 2.5-5 μg/ml	[171]
Terpene	Farnesol	MBC = 20 μg/mL	Cell membrane disturbance	*S. aureus*	• Caused the largest initial and total leakage of K^+^ ions between the tested terpene alcohols • These effects were dose-dependent	[174]
	Nerolidol	MBC = 40 μg/mL	Cell membrane disturbance	*S. aureus*	• After farnesol, caused the largest initial and total leakage of K^+^ ions between the tested terpene alcohols • These effects were dose-dependent	[174]
	Dehydroabietic acid	-	Cell membrane disturbance	*S. aureus*	• Midpoint antibacterial concentration (GD_50_) for 24 h incubation was < 20 μg/mL	[178]
	(4R)-(-)-carvone	-	Cell membrane disturbance	*C. jejuni*, *Enterococcus faecium, E. coli*	-	[182]
		-	Inhibits the transformation of cellular yeast form to the filamentous form	*C. albicans*	-	[173]
	(4S)-(+)-carvone	-	Cell membrane disturbance	*L. monocytogenes*	-	[182]
		-	Inhibits the transformation of cellular yeast form to the filamentous form	*C. albicans*	-	[173]
	Thymol	49.37 μg/ml	Inhibits H(+)-ATPase in the cytoplasmic membrane, cell membrane disturbance, efflux pump inhibition	*C. albicans*	• Exhibited synergistic activity in combination with fluconazole	[183]
		51.25 μg/ml		*C. glabrata*		
		70 μg/ml		*C. krusei*		
		200, 150, 125, 125, 400, 300, 100, 250, 500, 300, 450 μg/ml, respectively		*A. niger, A. fumigatus, A. flavus, A. ochraceus, Alternaria alternata, Botrytis cinerea, Cladosporium* spp*., Penicillium citrinum, P. chrysogenum, Fusarium oxysporum, Rhizopus oryzae*	• The most growth inhibition was related to *Cladosporium* spp.	[184]
		8, 10, 6.5, 5 μg/ml, respectively		*E. coli, E. aerogenes, S. aureus, P. aeruginosa*	• Antibacterial efficiency of free thymol was higher than its *in situ* activity within the *Thymus capitatus* leaves	[186]
		312 μg/ml		*Salmonella typhimurium, S. enteritidis, S. saintpaul*	• Reduced Biofilms of *Salmonella* spp. on polypropylene, but did not eliminate the adhered cells	[187-188]
	Carvacrol	50, 100, 100, 100, 350, 300, 100, 150, 125, 125, 200 μg/ml, respectively	Cell membrane disturbance, efflux pump inhibition	*A. niger, A. fumigatus, A. flavus, A. ochraceus, Alternaria alternata, Botrytis cinerea, Cladosporium* spp*., Penicillium citrinum, P. chrysogenum, Fusarium oxysporum, Rhizopus oryzae,*	• The most growth inhibition was related to *Aspergillus* spp.	[184]
		8, 8, 7, 7 μg/ml, respectively		*E. coli, E. aerogenes, S. aureus, P. aeruginosa*	• Antibacterial efficiency of free carvacrol was higher than its *in situ* activity within the *Thymus capitatus* leaves	[186]
		156 μg/ml		*S. typhimurium, S. enteritidis, S. saintpaul*	• Reduced Biofilms of *Salmonella* spp. on polypropylene, but did not eliminate the adhered cells	[187]
	Eugenol	400, 400, 450, 350, 500, 450, 350, 350, 400, 400, 350 μg/ml, respectively	Cell membrane disturbance	*A. niger, A. fumigatus, A. flavus, A. ochraceus, Alternaria alternata, Botrytis cinerea, Cladosporium* spp*., Penicillium citrinum, P. chrysogenum, Fusarium oxysporum, and Rhizopus oryzae*	• The most growth inhibition was related to *Cladosporium* spp. and *Rhizopus oryzae*	[184]
		2 μg/mL		*H. pylori*	• Maintain the bactericidal activity at low pH levels. The microorganism did not show any resistance to it even after 10 passages in the presence of sub-MIC levels	[191]
		0.04% V/V in MHB		MRSA, MSSA	• Inhibited biofilm construction • Interrupted cell-to-cell communication • Eradicated the pre-established biofilms • Killed the bacteria in biofilms	[192]
		150-300 μg/mL		*P. aeruginosa*	• Had anti-virulence, anti-biofilm and biofilm eradication properties • Could synergistically enhance bactericidal effect of gentamicin on biofilm associated bacteria	[193]
	Menthol	150, 150, 100, 100, 450, 400, 125, 100, 300, 200, 250 μg/ml, respectively	Cell membrane disturbance	*A. niger, A. fumigatus, A. flavus, A. ochraceus, Alternaria alternata, Botrytis cinerea, Cladosporium* spp*., Penicillium citrinum, P. chrysogenum, Fusarium oxysporum, Rhizopus oryzae*	• The most growth inhibition was related to *Cladosporium* spp. and *Aspergillus* spp.	[184]
	Cinnamaldehyde	2 μg/mL	Cell membrane disturbance	*H. pylori*	• Maintain the bactericidal activity at low pH levels. • The microorganism did not show any resistance to it even after 10 passages in the presence of sub-MIC levels	[191]
		0.25 μL/mL	Cell membrane and metabolic activity disturbance	*E. coli* and *S. aureus*	-	[194]
	Ursolic acid	-	Cell membrane disturbance	*E. coli*	-	[190]
	α-Amyrin	-	Cell membrane disturbance	*E. coli*	-	[190]

#### Alkaloids

**Fig. 3:**
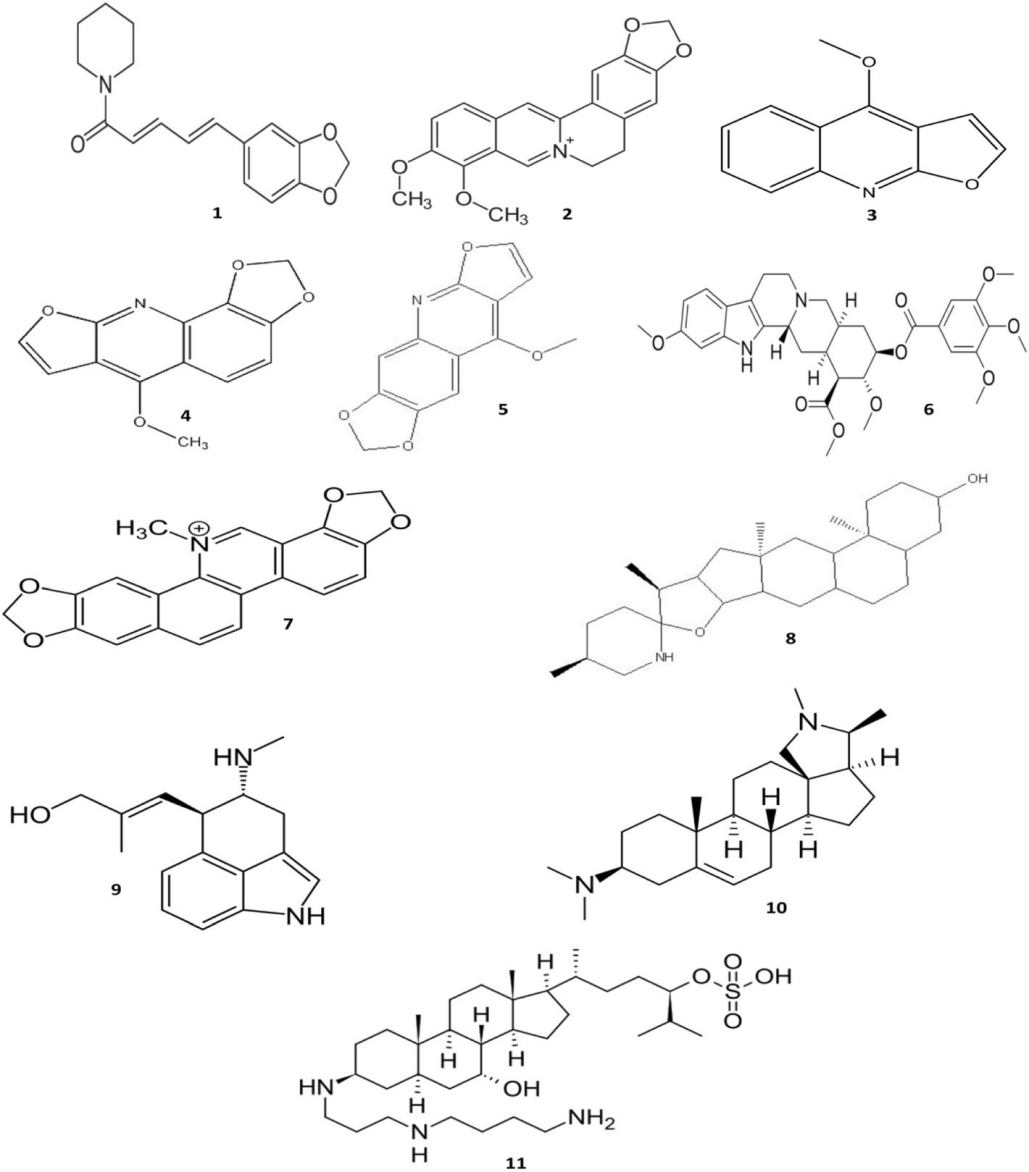
Chemical structures of selected antimicrobial alkaloids

Alkaloids are heterocyclic nitrogen compounds that contain extremely variable chemical structures. The antibacterial activity of alkaloids have been already proven and many studies have indicated that these compounds can play a significant role throughout the treatment of many infectious diseases [44]. Most of the alkaloids act through EPI activity, which stands as a putative mechanism of antibacterial functionality. The most important alkaloids with potent antibacterial activities have been illustrated in Fig. 3.

Piperine (1), a piperidine-type alkaloid, isolated from *Piper nigrum* and *Piper longum*, when co-administered with ciprofloxacin, inhibited growth of a mutant *S. aureus* and also the MIC values for *S. aureus* reduced markedly [45]. Co-administration of piperine and gentamicin was effective in combination MRSA infection [46]. The application of piperine as the EPI has been studied and the results showed that this compound affected the NorA EP activity of *S. aureus* and MRSA [46, 47].

Berberine (2) is known as an isoquinoline alkaloid and can be found in roots and stem-bark of Berberis species, which is also the main active ingredient of *Rhizoma coptidis* and *Cortex phellodendri* and has been widely used in traditional medicine. This compound has exhibited activity against bacteria, fungi, protozoa, and viruses. DNA intercalation, targeting RNA polymerase, gyrase and topoisomerase IV, and finally, the inhibition of cell division are the antibacterial action mechanism of berberine [48–50]. The results of another study have indicated that its antibacterial properties are related to the inhibition of cell division protein FtsZ [51]. This compound has been also able to inhibit the cell function of bacteria through various mechanisms such as damaging the cell structure, as well as protein and DNA synthesis inhibitors that result in bacterial death [52]. Up to the present, berberine has emerged as a strong antibacterial agent for the purpose of replacing conventional antibiotics and also to overcome the obstacles of antibiotic resistance. Ungeremine is another iso-quinoline alkaloid that had been obtained from the methanol extract of *Pancratium illyricum* L. bulbs, which has been observed to possess antibacterial activities. This compound can cause a remarkable increase in the DNA cleavage by targeting and inhibiting the bacterial topoisomerase IA [53, 54].

Quinoline alkaloid, such as dictamnine (3) [55], kokusagine (4) and maculine (5) that had been isolated from the stem bark of *Teclea afzeli* has exhibited promising antibacterial activity [56]. Natural or synthetic quinolone alkaloids can inhibit type II topoisomerase enzymes and consequently, inhibit the DNA replication as well [57]. Alkyl methyl quinolones can reduce the O_2_ consumption in the treated bacteria and accordingly, be considered as respiratory inhibitors [58].

Reserpine (6) is an indole alkaloid that had been procured from *Rauwolfia serpentina* and well known natural compound with potent EPI activity [59]. A wide range of bacterial species, including *Staphylococcus spp.*, *Streptococcus spp.*, and *Micrococcus spp.*, has shown an enhanced antibiotic susceptibility upon their co-administration with reserpine [60]. In addition, reserpine is capable of remarkably enhancing the susceptibility of MDR isolates that belong to *A. baumannii* towards antibiotics. It should be also noted that the AdeABC EPs have been over-expressed throughout this clinical isolates [61]. It has been indicated in another study that the overexpression of EP has been the main resistance mechanism to fluoroquinolones in resistant *Stenotrophomonas maltophilia*, while the addition of reserpine had decreased the antibiotic resistance [62]. Taken together, it can be concluded that reserpine is a compound with potent EPI activity in both Gram-positive and -negative bacteria.

As it has been mentioned previously, FtsZ inhibitors could be considered as a novel class of antibacterial agents with the potential of exhibiting broad-spectrum activity. Some of the naturally occurring alkaloids such as sanguinarine and berberine are known to be capable of altering the functionality of FtsZ [63].

Sanguinarine (7) can be obtained through the extraction of some particular plants such as *Chelidonium majus*, *Sanguinaria canadensis*, and *Macleaya cordata*. In a study, the activity of sanguinarine against MRSA strains has been tested and its mechanism of action has been also investigated. It was shown that the treatment of bacteria with this compound can lead to the release of membrane-bound cell wall autolytic enzymes and results in the lysis of cell; on the another hand, the transmission electron microscopy of MRSA has shown alterations throughout the formation of septa. Taken together, the possible action mechanism of sanguinarine against MRSA has been suggested to be compromising the cytoplasmic membrane [64]. In the previous study, it has been indicated that next to being a strong DNA intercalator, sanguinarine and berberine are potent replication and transcription inhibitors [65]. It has been also suggested that sanguinarine can exhibit antimycobacterial activities against two model species of mycobacteria, which include *Mycobacterium aurum* and *Mycobacterium smegmatis* [66].

Tomatidine (8) is a steroidal alkaloid that is procured from solanaceous plants including tomato, potato, and eggplant, which has displayed potent antibacterial activity against *S. aureus* alone or in combination with aminoglycosides [67]. Additionally, the synergistic effects between tomatidine and aminoglycosides against drug-resistant strains of *S. aureus* have been proven as well [68]. Tomatidine could be considered as a potential antibiotic potentiator for different antibiotics, such as gentamicin, cefepime and ciprofloxacin, and ampicillin, against both Gram-positive and -negative bacteria that include *S. aureus*, *P. aeruginosa*, and *Enterococcus faecalis* infections [69].

Chanoclavine (9) has been categorized as a tricyclic ergot alkaloid that is isolated from *Ipomoea muricata* and has exhibited synergistic effects upon being co-administered with tetracycline against MDR *Escherichia coli*. This compound has been discovered to inhibit EP, which seems to be related to the ATPase-dependent ones [70].

*Holarrhena antidysenterica* belongs to the Apocynaceae family and has been traditionally employed for the treatment of different diseases such as dysentery, diarrhea, fever, and bacterial infections [71]. *H. antidysenterica* barks are composed of alkaloids, particularly steroidal alkaloid conessine (10) that is responsible for its therapeutic effects [72]. This compound is effective against both Gram-positive and -negative bacteria and has displayed potential antibacterial activity. Results of the available studies have indicated that the antibacterial activity of conessine is almost similar to that of antibiotics that had been used as control. Moreover, the combination of conessine with conventional antibiotics has exerted synergistic effects [73, 74]. This compound has been also utilized as the resistance-modifying agents in regards to the susceptibility of *A. baumannii* towards antibiotics. Furthermore, significant synergistic activities have been observed as a result of the combination of conessine with antibiotics. Additionally, this substance has displayed EPI activity against *AdeIJK* EP, which plays an important role in effluxing the multiple antibiotics in *A. baumannii* [75, 76].

Squalamine (11) is a natural steroid-polyamine compound that had been isolated for the very first time from the dogfish shark. However, this compound, which is not mainly found in plants, has shown broad-spectrum antimicrobial activity by disrupting the microbial membranes and influencing their permeability. In Gram-negative bacteria, squalamine interacts with the negatively charged phosphate groups in the bacterial outer membrane, which is the first step of the sequences that lead to the disruption of membrane. However, in the case of Gram-positive bacteria, it can cause the depolarization of the cytoplasmic membrane, resulting in the leakage of cytoplasmic contents and lead to the rapid death of cells [77].

#### Organosulfur compounds

**Fig. 4:**
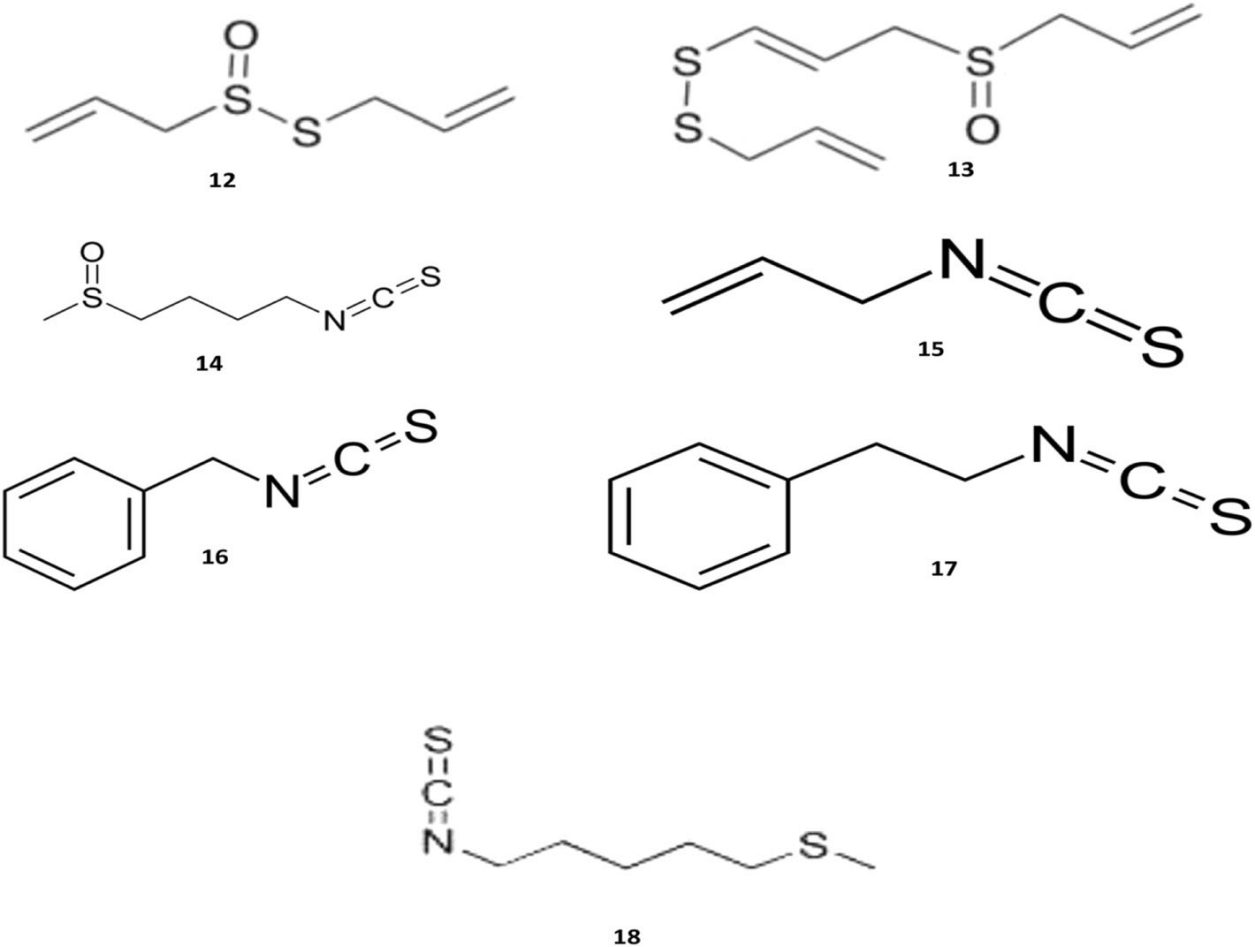
Chemical structures of selected antimicrobial organosulfur compounds

There is an extensive number of reports in the literature on the topic of antibacterial and antifungal activities of sulfur-containing compounds that are obtained from plants [78, 79]. Sulfur-containing compounds such as allicin, ajoene, dialkenyl, and dialkyl sulphides, S-allyl cysteine and S-ally-mercapto cysteine, and isothiocyanates have exerted antibacterial activities against both Gram-positive and -negative bacteria [43, 80]. It has been discovered through the performed investigations that the plants with high concentrations of polysulphides are capable of displaying a wide spectrum of antimicrobial activity [81, 82]. The most important compounds with potential antibacterial activities are shown in Fig. 4.

Allicin (12), also known as diallyl thiosulfinate, is an organosulfur compound that is obtained from garlic (*Allium sativum*), a species in the family *Alliaceae*. The antimicrobial activities of this compound have been acknowledged for a long time and its antibacterial activities have been observed against a wide range of bacteria such as *Staphylococcus epidermidis*, *P. aeruginosa*, *Streptococcus agalactiae*, MRSA, and oral pathogens that can cause periodontitis [83]. Another study has confirmed the fact that allicin can potentiate the antibacterial activities of some antibiotics including cefoperazone, tobramycin, and ciprofloxacin, against *P. aeruginosa* [84].

The mechanism of allicin antimicrobial activity has been reported, which had been due to the inhibition of sulfhydryl-dependent enzymes including alcohol dehydrogenase, thioredoxin reductase, and RNA polymerase [85]. These observations have been confirmed by detecting the reduced inhibitory effects of allicin that had been caused through the addition of cysteine and glutathione to the media. These two compounds are able to react with allicin disulfide bond and result in preventing the microbial cells damages [86]. Furthermore, allicin has been detected to partially inhibit the DNA and protein synthesis as well. The immediate effect of allicin on RNA has been also proven, which indicates the possibility of RNA is being a possible target of allicin [87].

Ajoene (13) is another organosulfur compound that has been found in the extracts of garlic. This compound consists of a mixture of two main stereoisomers that include E- and Z-ajoene. Ajoene has displayed broad-spectrum antimicrobial activities against both Gram-positive and -negative bacteria, fungi, and protozoa; however, it has exhibited more potent antiviral activity when compared with allicin. In addition, and similar to allicin, the inhibitory effect of ajoene has been greatly reduced upon the appending of cysteine, which had been due to the existing interaction between an amino acid and disulfide bonds of the compound. Up to this point, it can be concluded that ajoene contains the same antibacterial mechanism of action to that of allicin, which had functioned in accordance with different thiol-dependent enzymatic systems [88].

Isothiocyanates (ITCs) are volatile organosulfur compounds that have been obtained through the reaction between plant glucosinolates and myrosinase enzyme. Subsequent to tissue disruption, the enzyme hydrolyzes into active compounds such as nitriles, thiocyanates, and ITCs. These compounds have been exclusively discovered throughout the order of Capparales and exist abundantly in the plants of Brassicaceae family such as cauliflower, cabbage, mustard, and broccoli. Among all of them, ITCs have displayed more potent inhibitory effects on a variety of pathogenic bacteria, which has labeled them as promising antibacterial candidates.

The antimicrobial activity of ITCs, which are extracted from horseradish (*Armoracia rusticana*) root, have been evaluated against oral pathogens and the obtained results have indicated that these compounds are capable of exhibiting the strongest antimicrobial activities [89]. ITCs have been observed to be potently bactericidal against *Helicobacter pylori*, which acts by inhibiting the urease and reducing the inflammatory component of Helicobacter infections [90].

Although the antimicrobial mechanisms of ITCs have not been completely comprehended, yet it has been estimated that its antimicrobial activity might be related to the reactivity of ITCs with proteins, which can disturb the in vivo biochemical processes. The carbon atom of ITC group (−N=C=S) is highly electrophilic and reacts easily with amines, thiols, and hydroxyls; consequently, they can readily attack thiols and the amines of amino acid residues that exist within proteins, yet they mainly attack the sulfhydryl groups [91]. Cysteine plays a crucial role in protein structure, regulatory function and also protein stabilization with different mechanisms. ITCs are known to inhibit ATP binding sites of P-ATPase in bacteria (*E. coli*), which is performed by attacking the cysteine residue [92].

Sulforaphane (14) is a compound that exists within the ITCs and can be found in various plants such as *Diplotaxis harra*. It is derived from 4-methyl sulfinyl butyl glucosinolate and has displayed potent anticarcinogenic and antibacterial activities especially against *H. pylori*, which is known as a potential causal agent of stomach cancer. This substance has been also effective against *S. aureus* and *Listeria monocytogenes*; therefore, sulforaphane could be considered as a good candidate for functioning as a novel natural antibacterial agent [93].

Allyl ITCs (15) AITCs, is an organosulfur compound with the formula of C=C-C-N=C=S, which has been detected to show potent antibacterial activities. It is often found in the plant of *Brassicaceae* family such as *Armoracia rusticana* and *Eutrema japonicum* [94]. Throughout the study, the antibacterial properties of AITC against *E. coli* and *S. aureus* have been assessed, which had resulted in proving its bacteriostatic and bactericidal activities [94]. Next to being effective in reducing the MIC values of erythromycin against *S. pyogenes* [95], AITCs has also displayed synergistic effects with streptomycin against *E. coli* and *P. aeruginosa* [96]. It has been indicated in another research that AITC had exhibited low inhibitory effects against three Gram-positive bacteria [55]. Various mechanisms have been reported for the antimicrobial activities of AITC. Upon being utilized as a vapor, this compound can damage the cell wall integrity and lead to the leakage of cellular metabolites. Nevertheless, the treatment has provoked internal structure changes, which had been observed through the usage of electron microscopy [55, 97, 98]. Delaquis and Mazza suggest that AITC might cause inactivation of essential intracellular enzymes by oxidative cleavage of disulfide bonds [99]. Lin and co-workers have reported the observed induced damages on the bacteria cell after being exposed to AITC, which had resulted in the creation of pores on the cell membranes and caused the leakage of intracellular substances [55].

Benzyl ITCs (16) (BITC), is an isothiocyanate that can be found in *Alliaria petiolate* [100]. This substance has been evaluated against 15 isolates of MRSA and has been perceived to be bactericidal towards 11 of them. Based on this observation, BITC can be effective in suppressing the MRSA strains [101]. The potent antibacterial activity of this compound is apparently dependent on chemical structure. BITC has both lipophilic and electrophilic properties and, it can penetrate through the outer bacterial membrane and disturb the ability of bacterium for maintaining the membrane integrity, which is similar to what has been found in regards to cationic peptides [102].

Phenethyl isothiocyanate (17) (PEITC), is another type of ITCs that can be found within brassica vegetables such as *Brassica campestris* and *Brassica rapa* [103]. This compound has been used to evaluate the in vitro antibacterial activity against bacteria that had been isolated from the human intestinal tract. Although PEITC has exhibited potential antimicrobial activities against Gram-positive bacteria, however, it has demonstrated low inhibitory activity against the Gram-negative ones [104]. The antifungal activities of PEITC against *Alternaria brassicicola* has been also studied and promising results have been observed [105]. The putative anti-fungal properties of ITCs might be related to different factors including the decreased rate of oxygen consumption, intracellular accumulation of reactive oxygen species (ROS), and finally the depolarization of mitochondrial membrane [106].

Berteroin (18) which exists in broccoli (*Brassica oleracea L.*), has displayed the lowest MICs values against both extra- and intracellular bacteria and therefore, can be considered as an active compound with high bactericidal activity. In addition, this compound has been detected to be effective against *H. pylori* [107, 108].

#### Phenolic compounds

**Fig. 5:**
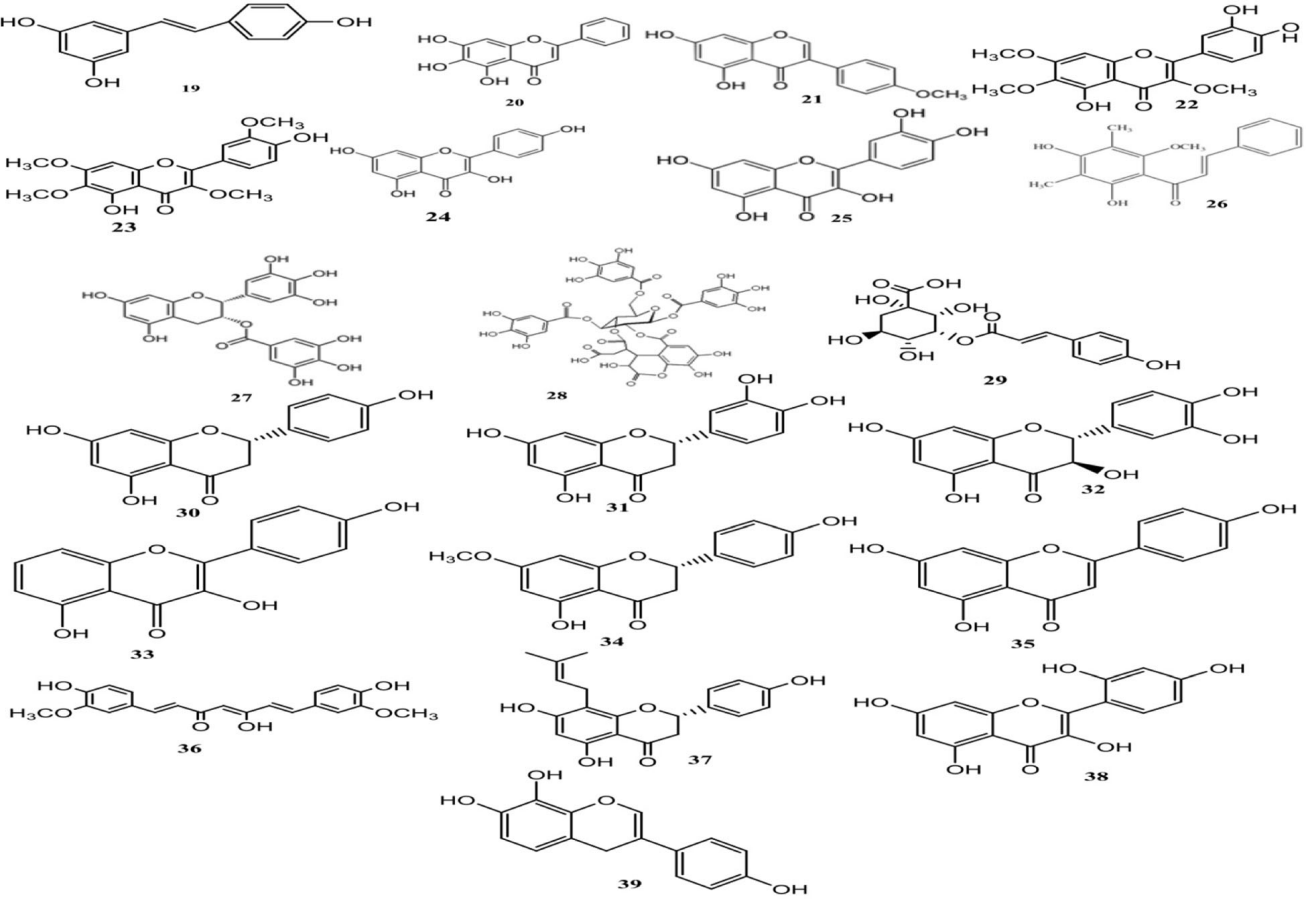
Chemical structures of selected antimicrobial phenolic compounds

Phenolic compounds include a wide range of bioactive natural compounds that are extensively utilized for medical purposes. These compounds, as bioactive molecules, play an important role in enhancing antibiotic activity against resistant pathogens through various mechanisms [109–111]. Figure 5 demonstrates the most important phenolic compounds with potential antibacterial activities.

Reducing the EP activity and acting as EPIs strand as some of the most significant mechanisms. These types of compounds have shown promising EPI activity against pathogenic bacteria. Table 1 contains the list of the most important EPIs that have been isolated from plant source.

Resveratrol (19) is known as a natural phenolic compound that has exhibited EPI activity against different bacteria, which is capable of inhibiting the activity of *CmeABC* EPs of *Campylobacter jejuni* or EPs of *M. smegmatis* [112, 113]. Ferreira et al. studied the EPI activity of this compound against *Arcobacter butzleri* LMG 10828 and *Arcobacter cryaerophilus* LMG 10829. The results indicated an increase in the accumulation of ethidium bromide in the presence of resveratrol [114].

Baicalein is a flavone (20) isolated from the roots of *Thymus vulgaris*, *Scutellaria baicalensis,* and *Scutellaria lateriflora*. In previous studies, the antibacterial activity of *Scutellaria baicalensis* extract has been reported [115]. The antimicrobial activities of different extracts of *S. litwinowii* have been screened against the standard strains of *S. aureus*, *Bacillus cereus*, *P. aeruginosa*, *E. coli*, and *C. albicans*. The minimum inhibitory and bactericidal concentrations have been determined through the means of broth microdilution method and tetrazolium chloride salt. The results of this investigation indicated that extracts obtained from the aerial parts of *S. litwinowii* possessed antioxidant and antimicrobial properties [116]. Baicalein could also remarkably restore the effectiveness of β-lactam antibiotics, tetracycline, and ciprofloxacin against MRSA via inhibition of the NorA EP [117]. Furthermore, synergistic effects have been observed as a result of the combination of baicalein with tetracycline against *E. coli* by the inhibition of EP [118].

The inhibitory activity of biochanin A (21), an isoflavone, on efflux system of MRSA has been previously studied, and the results had indicated that this substance could inhibit MRSA EPs by reducing the expression of *NorA* protein [119]. The Inhibitory effect of biochanin A on intracellular bacteria of Chlamydia spp. has been investigated and the outcomes had suggested that this compound is a potent inhibitor of *Chlamydia* spp. [120]. Moreover, the potent EPI activity of biochanin A has been demonstrated against *Mycobacterium* strains [113, 121]. Several other flavonoids also showed inhibitory activity against NorA EP. Chrysosplenol-D (22) and chrysoplenetin (23), which are two methoxylated flavones from *Artemisia annua* have inhibited NorA EP in the presence of subinhibitory concentrations of berberine as a substrate of NorA EP [122]. Isoflavonoids and flavonolignans are two other classes of phenolic compounds that could inhibit NorA and increase the potency of norfloxacin and berberine [123]. Silybin, a flavonolignan from the famous medicinal plant *Silybum marianum* and isoflavonoids biochanin A, genistein, and orobol from *Lupinus argenteus* potentiated *S. aureus* against many substrates of NorA EP [123, 124].

The topic of hybridization of antibiotics with flavonoid has attracted the attention of many researches since it can reduce the EPs activity [125]. It has been indicated that the antibiotic accumulation and activity of hybrid molecules have been remarkably enhanced, which confirms the desired dual mode of action.

Kaempferol (24) is an active flavonoid that has been considered as a potential candidate against different pathogenic microbes since its effectiveness against fluconazole-resistant *C. albicans* and also MRSA has been proven [126, 127]. The EPI activity of kaempferol against MRSA was similar to that of verapamil, as a control, and mainly due to the effect on the NorA pump. Kaempferol rhamnoside a natural glycoside derivative of kaempferol from *Persea lingue* could increase antimicrobial activity of ciprofloxacin in a NorA overexpressed *S. aureus* strain up to 8-folds [128].

Brown et al. developed a new LC-Mass-based method for identification of EP inhibitors and studied pure flavonoids as inhibitors through the means of this novel approach. Interestingly, they have discovered that although the fluorescence-based technique has been unable to correctly exhibit the inhibitory activity, yet in the course of the LC-Mass-based method, rhamentin and kaempferol have displayed fine inhibitions with the IC_50_ values of 66 and 60 μM, respectively [129]. In addition, quercetin (25) has displayed a moderate inhibition of EP while in previous studies, there have not been any signs of inhibitory activity. Apparently, it can be stated that flavonoids possess quenching effects when positioned in the fluorescence-based methods, which can affect the results that are achieved throughout this type of studies [129].

Chalcones are other groups of phenolic compounds that can inhibit EP and increase the activity of antibiotics. 4′,6′-Dihydroxy-3′,5′-dimethyl-2′-methoxychalcone (26) isolated from *Dalea versicolor* inhibited NorA EP and reduced MIC of erythromycin from 0.4 to 0.1 μg/mL [130]. A more comprehensive work on chalcones has been performed by Holler et al. Screening of a library of 117 natural and synthetic chalcones revealed that two synthetic chalcones namely 4-phenoxy-4′-dimethylaminoethoxychalcone and 4-dimethylamino-4′-dimethylaminoethoxychalcone were equipotent to reserpine, an alkaloid with potent NorA EP inhibitory activity [131]. In accordance with this study, chalcones could be considered as candidates for more clinically relevant researches in the future to overcome this type of antibiotic resistance.

The catechin gallates such as epigallocatechin gallate (EGCG) (27) are another group of phenolic compounds that provide health benefits and also exhibited potent antimicrobial activity against resistant pathogens such as MRSA. These compounds were found to weakly inhibit the NorA EP [132].

Antimicrobial activity of natural phenolic compounds is not limited to their ability to inhibit efflux pump. Several phenolic compounds with diverse mechanisms of action have been identified to date [109]. The inhibition of DNA gyrase can be stated as an example of these mechanisms that has led to the introduction of clinically approved aminocoumarin antibiotic novobiocin [133]; nevertheless, since these types of compounds have not been produced by plants, they had been considered out of the scope of this review.

Green tea polyphenols (tannins) [134], chebulinic acid [135] and anthraquinones [136] are natural phenolic compounds that showed inhibitory activity against DNA gyrase. EGCG from green tea can inhibit the B subunit of DNA gyrase at its ATP binding site [134]. Owing to the multiple modes of EGCG action, which include the inhibition of EP, as well as the inhibition of chromosomal penicillinase and DNA gyrase, this natural polyphenol stands as one of the special interests for future researches. Chebulinic acid (28) is another tannin that had been initially isolated from *Terminalia chebula*. The virtual screening of several natural compounds revealed that chebulinic acid can inhibit quinolone resistant mutants of *M. tuberculosis* DNA gyrase effectively [135]. However, the findings were limited to in silico studies and no in vitro experimental study was done. Future in vitro studies should be done to unravel the significance of chebulinic acid as a DNA gyrase inhibitor and antituberculosis agent.

Haloemodins are semisynthetic natural anthraquinone derivatives that can strongly inhibit DNA gyrase in MRSA and vancomycin-resistant *Enterococcus faecium*. Several halogenated analogous of natural product emodin have been synthesized and showed potent activity against bacterial DNA gyrase, while weak activity was observed against human topoisomerase I [136].

The novel phenolic compound isolated from *Cedrus deodara*, 3-p-trans-coumaroyl-2-hydroxyquinic acid (CHQA) (29), has shown potent antibacterial activity against eleven food-borne pathogens. The elucidated mechanism of action of CHQA against S. aureus (MIC values ranging from 2.5–10 mg/mL) is causing damage to the cytoplasmic membrane and inducing the leakage of intracellular constituents that is due to the occurrence of significant membrane hyperpolarization with a loss of membrane integrity, which had been determined through the usage of membrane potential measurements and flow cytometric analysis. Authors believed that CHQA may be a candidate to serve as a natural antimicrobial agent for the food industry [137]. It seems that generally hydroxycinnamic acids (p-coumaric, caffeic and ferulic acids) natural products are capable of interfering with membrane integrity, while p-coumaric has the most interfering activity in this group owing to its more lipophilic nature [138]. In a study on the effects of phenolic compounds on wine lactic acid bacteria (*Oenococcus oeni* and *Lactobacillus hilgardii*) coumaric acid, a hydroxycinnamic acid compound showed the most activity [138].

A comprehensive structure-activity relationship (SAR) study on membrane interaction effects of flavonoids has proved that the antibacterial activity of flavonoids has a positive correlation with their ability to rigidify the *E. coli* membrane, which suggests that one possible mechanism of action of flavonoids is reducing membrane fluidity. Kaempferol with the higher CLogP and the positive charge on C3 displayed the most potent activity against *E. coli* [139]*.*

Phenolic compounds can also interact with some crucial enzymes which are responsible for the production of precursors of bacterial cell membrane including beta-Ketoacyl acyl carrier protein synthase (KAS) II and III or enzymes that were involved in elongation cycle of fatty acid biosynthesis including FabG, FabI, and FabZ. An in silico screening of flavonoids that had been performed for assessing their binding affinity to E. faecalis KAS III enzyme has indicated that flavanones (naringenin (30), eriodictyol (31), and taxifolin (32)) are more potent inhibitors, while the results of the in vitro studies have displayed a moderate antibacterial activity against E. faecalis and vancomycin resistance strain [140]. However, another study on *E. coli* has revealed that 3,6-dihydroxyflavone (33) (a flavonol) can also bind potently to KAS III and KAS I and its MIC was 512 μg/mL, indicating that many classes of flavonoids can bind to KAS enzymes [109].

EGCG from green tea could inactivate beta-ketoacyl-[acyl carrier protein] reductase (FabG) in *E. coli* via covalent binding to the protein that leads to aggregation of FabG [141]. Sakuranetin (34) a methoxy derivative of the flavanone naringenin from *Polymnia fruticose* competitively inhibited FabZ in *H. pylori* and the MIC value of sakuranetin against *H. pylori* was 87.3 μM [142]. Although quercetin and apigenin (35) have been also capable of inhibiting the FabZ, yet their inhibitory activity has been lower than that of sakuranetin, whereas quercetin has displayed lower MIC values.

Curcumin (36) is a well-known compound that is obtained from Tumeric and the recent studies have revealed that it can display bactericidal activity by damaging the cell membranes of *S. aureus* and *E. coli*. The authors have related the observed activity to the amphipathic and lipophilic chemical structure of curcumin that can penetrate to membrane bilayer and enhance its permeability [143]. d-Alanine:d-alanine ligase is a critical enzyme in the assembly of peptidoglycan precursors of the cell wall. Quercetin and apigenin, two abundant flavonoids, are the phenolic compounds that could inhibit d-alanine:d-alanine ligase in *H. pylori* and *E. coli*. Quercetin was more active (48.5 and 19.9 μM, respectively) than apigenin (132.7 and 163 μM, respectively) and both were reverse inhibitors and competitive with ATP [144]. However, the MIC values for both strains were high which reflects the low inhibitory activity of these compounds.

Some other phenolic compounds can directly interact with peptidoglycan and inhibit cell wall biosynthesis. Sophoraflavanone B (37) is a prenylated flavonoid that showed MIC of 15.6–31.25 μg/mL against MRSA while detailed studies revealed its direct interaction with peptidoglycan as the possible mechanism of action [145].

The inhibition of some enzymes including dihydrofolate reductase, urease, and sortase were likewise proposed as a mechanism of action of some of the phenolic compounds [146–148]. Inhibition of dihydrofolate reductase was identified as one of the diverse mechanisms of action of EGCG against 18 clinical isolates of nosocomial pathogen *Stenotrophomonas maltophilia*. This mechanism was similar to the well-known blocker of dihydrofolate reductase and as the MIC values were very low in some isolates (4 μg/mL) the authors suggest that by more clinical studies, trimethoprim can be replaced with EGCG in patients that cannot tolerate the side effects of trimethoprim [148].

A study on the inhibitory activity of *Curcuma longa* against sortase A from *S. aureus* has revealed that curcumin is a potent inhibitor of this enzyme with the IC_50_ of 13.8 μg/mL, while an IC_50_ of 40.6 μg/mL has been detected in the case of p-hydroxy mecuri benzoic acid as the positive control. Although demethoxycurcumin and bisdemethoxycurcumin have been also enabled to inhibit sortase A, yet their activity has been lower than curcumin [149]. In a similar fashion, a bioassay-guided study on the inhibitory activity of compounds from barks of *Rhus verniciflua* against *S. aureus* sortase identified morin (38) (a flavonol) as a potent inhibitor of sortase A and B with IC_50_ values of 37.39 and 8.54 μM, respectively. Although the flavonols were unable to inhibit the growth of bacteria, more studies indicated that flavonols have fibrinogen cell-clumping activity [150].

Some specific phenolic compounds are also considered to be potent inhibitors of urease as a major virulence factor of *H. pylori*. A SAR survey that had been performed by Xiao et al*.* have revealed that the 4-deoxy analogues of flavonoids are potent inhibitors of urease and 4′,7,8-trihydroxyl-2-isoflavene (39) has inhibited the enzyme with the IC_50_ of 0.85 μM which is 20-fold stronger than acetohydroxamic acid as a well-known urease inhibitor. Considering the side effects that had been observed from synthetic inhibitors of urease throughout the in vivo studies, apparently 4′,7,8-trihydroxyl-2-isoflavene can be a promising candidate for future in vivo investigations [147].

Phenolic compounds showed diverse mechanisms of action against different bacterial strains from synergistic activity via inhibition of EPs, interacting with the cell membrane and inhibition of cell wall biosynthesis to inhibition of certain critical enzymes including urease, sortase A and dihydrofolate reductase. The observed activities were remarkable in some studies making phenolic compounds a good candidate for future in vivo studies and even clinical trials. EGCG and curcumin are good examples of such compounds that can act with various mechanisms of action thus can unable bacteria to simply become resistant to the treatment.

#### Coumarins

**Fig. 6:**
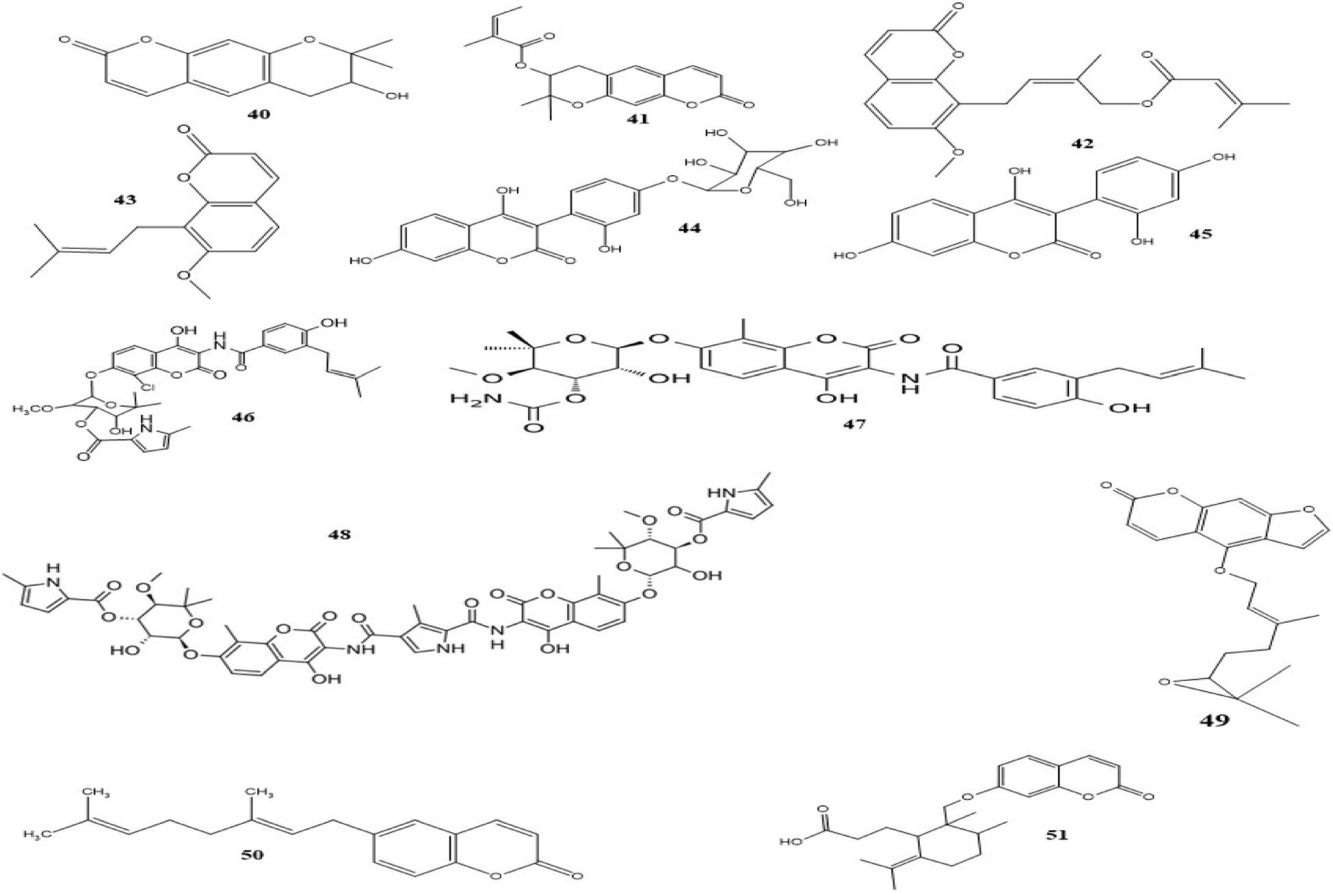
Chemical structures of selected antimicrobial coumarins

Coumarins are produced naturally by many plants and microorganisms [151]. Up to now, several bioactivities of coumarins have been reported including vasodilator, estrogenic, anticoagulant, analgesic, anti-inflammation, sedative and hypnotic, hypothermic, anti-helminthic, anti-cancer, antioxidant and dermal photosensitizing activity [152–154]. Figure 6 illustrates the well-known coumarins that have displayed high potential antibacterial activities.

Many reports have shown the antimicrobial activity of both natural and synthetic derivatives of coumarins [151, 155, 156]. For instance, Basile et al. reported various coumarins and pyranocoumarins extracted from the roots of *Ferulago campestris*, and the antibacterial and antioxidant activities of the most plentiful ones (agasyllin, grandivittin and aegelinol benzoate) against both Gram-negative and Gram-positive bacteria. Particularly, aegelinol (40) and agasyllin (41) were more active against ATCC strains of *Salmonella enterica* serovar *Typhi, Enterobacter aerogenes, Enterobacter cloacae*, and *S. aureus* (MIC = 16 μg/mL for aegelinol and 32 μg/mL for agasyllin). Both compounds also showed antibacterial activity against *Helicobacter pylori* in a dose-dependent manner between 5 and 25 μg/mL [157].

Tan et al. found one new and nine known prenylated coumarins from the root extract of *Prangos hulusii* and assessed the antimicrobial activity of the dichloromethane extract against both standard and clinical isolates. Although the new coumarin, 4′-senecioiloxyosthol (42) was the most active compound against *Bacillus subtilis* (MIC = 5 μg/mL), osthole (43), one of the previous extracted coumarins, presented acceptable antibacterial effect against more pathogens including *B. subtilis*, *S. aureus*, *Klebsiella pneumonia* and methicillin-sensitive *Staphylococcus aureus* (MSSA) (all MICs = 125 μg/mL) [158].

Screening the antimicrobial effect of six coumarins, which are common constituents of seven plants grown in Finland displayed that while the antibacterial and antifungal activities of these natural coumarins were generally weak, they were active against the fungal pathogen *Fusarium culmorum* [159]. In 2006, El-Seedi found a new aryl coumarin glucoside, asphodelin A 4′-*O*-*β*-D-glucoside (44) as well as its aglycon, asphodelin A (45) from *Asphodelus microcarpus.* In vitro antimicrobial evaluation was done against five microorganisms including *S. aureus, E. coli*, *P. aeruginosa*, *C. albicans,* and *Botrytis cinerea.* In general, asphodelin A exhibited more potent activity with MIC value ranging from 4 to 128 *μ*g/mL [160].

Maxwell investigated the SAR of three compounds with coumarin structure, clorobiocin (46), novobiocin (47) and coumermycin A1 (48) which all have derived from different *Streptomyces* species and displayed antibiotic activity. He concluded that there is an individual noviosyl sugar moiety in the chemical structure of each compound that is essential for biological activity, besides the coumarin portion. He also introduced coumarins as potent inhibitors of DNA topoisomerase type II, known as DNA gyrase [161].

Study on the SAR of coumarins revealed that both lipophilic characteristics, as well as a planar structure, are necessary for high antibacterial effects [155]. Indeed, since it seems that the antimicrobial activity of coumarins is due to the passive diffusion mechanism, these characteristics can facilitate cellular penetration especially for Gram-positive bacteria Moreover, Sardari et al. suggested that a free 6-OH and 7-OH in the coumarin nucleus play an important role for antifungal and antibacterial activity, respectively [162]. In another hand, systemic analysis of the SAR has indicated that coumarins with a methoxy function at C-7, besides a hydroxyl moiety at either C-6 or C-8 invariably have an antibacterial effect against a broad spectrum of bacteria. The existence of an aromatic dimethoxy arrangement, in turn, produces promising compounds against microorganisms with special growth factors requirements including *Haemophilus influenza*, beta-hemolytic *Streptococcus* and *Streptococcus pneumonia* [155]. Moreover, recent studies have suggested that coumarins are able to suppress the quorum-sensing network of bacterial pathogens and affect their ability in the development of biofilm formation and virulence factors production [153, 163–167].

Some coumarin derivatives were also able to inhibit EP in MSRA strain. Bergamottin epoxide (49) a furanocoumarin from *Citrus paradisi* (grapefruit) resulted in the 20-fold reduction in the MIC value of norfloxacin against MRSA, but not against MSSA, via inhibition of EP [168]. In another study on EP inhibition of seven coumarins from *Mesua ferrea*, two compounds showed inhibition against the EP system in MRSA and clinical isolates of *S. aureus* by the 8-fold reduction in the MIC of norfloxacin [169].

Coumarins are capable of binding to isoprene units in plant cells to form more complex structures. 6-Geranyl coumarin (50) and gallbanic acid (51) are two terpenoid coumarins that inhibited EP in *S. aureus*, significantly [170, 171]. Up to 8-fold reduction in the MIC of ciprofloxacin was observed for galbanic acid, and its mode of action and potency were comparable to verapamil as a well-known inhibitor of EP.

#### Terpenes

**Fig. 7:**
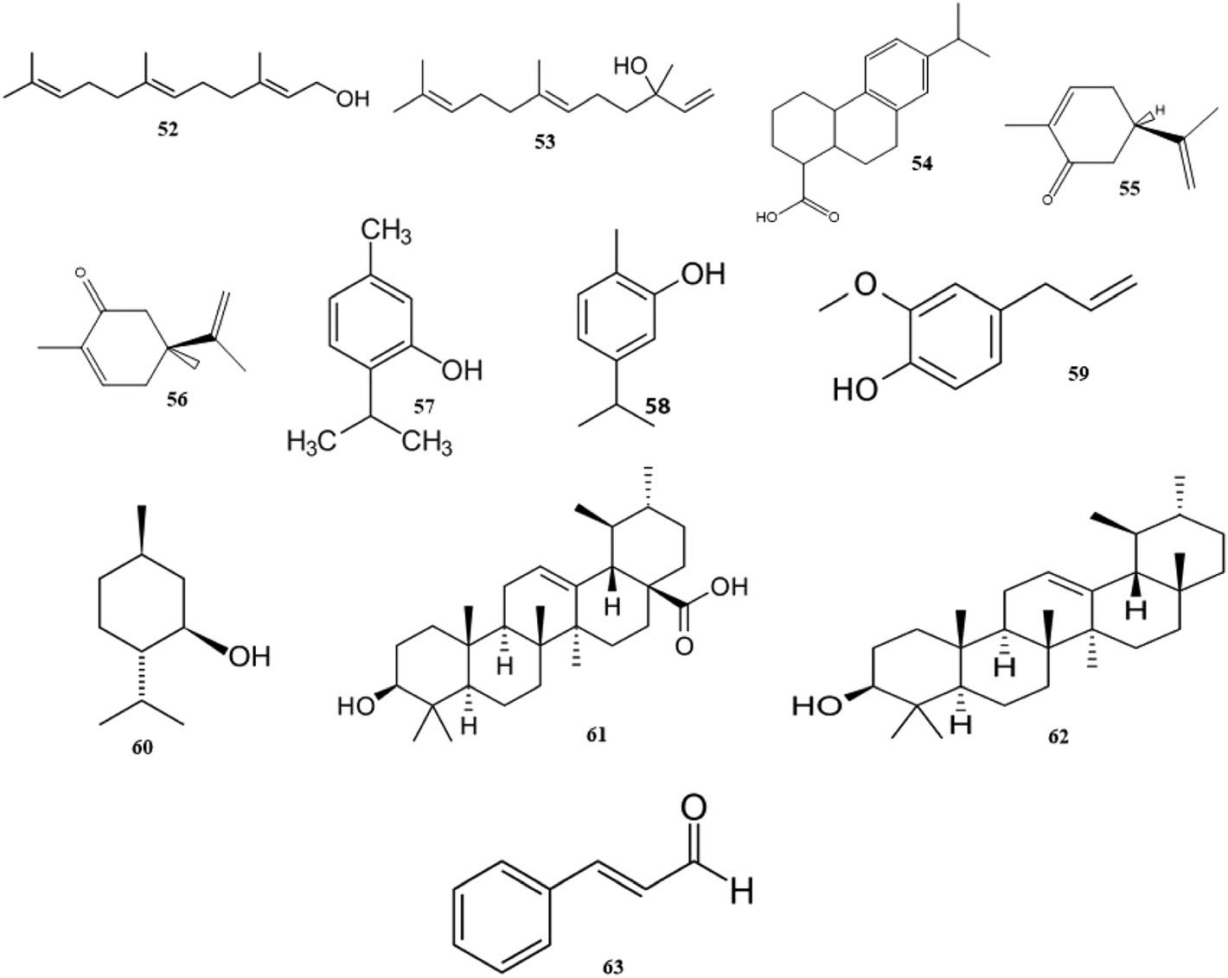
Chemical structures of selected antimicrobial terpenes

Terpenes or isoprenoids are considered as the most diverse family of natural products. They are widely outspread in nature, present in nearly all forms of life and perform numerous functions ranging from participation in the primary structure of cells (cholesterol and steroids in cell membranes) to contribution to the cell functions (retinal in vision, carotenoids in photosynthesis, quinones in electron transport) [172, 173]. They also exist abundantly in flowers, fruits, and vegetables. Especially, they can be found in high concentration in reproductive structures and foliage of plants, throughout and immediately following flowering. Terpenes are the major ingredients of herbal resins and responsible for the common fragrance of various plants [173]. It has been shown that several terpenes and their derivatives act as an imperative defense against herbivores and pathogens [173–177]. The different plant terpenes with high potential antibacterial activities are presented in Fig. 7.

Commonly, Gram-positive bacteria are more susceptible to terpenes than Gram-negative ones. The antimicrobial mechanism of terpenes is closely associated with their lipophilic features. Monoterpenes preferentially impact on the structures of the membrane through increasing its fluidity and permeability, altering the topology of its proteins and making disturbances across the respiration chain [173].

Togashi et al. investigated the inhibitory effects of various terpene alcohols with different aliphatic carbon chains including linalool (C6), geraniol (C8), nerolidol (C10), plaunotol (C11), farnesol (C12), and geranylgeraniol and phytol (C16) (numbering is from the first carbon bonded to the hydroxyl group) on the growth of *S. aureus*. Among all of the tested compounds, only farnesol (52) and nerolidol (53) displayed strong antibacterial effect with MBC as 20 and 40 μg/mL, respectively. They also examined the interaction of these terpene alcohols with bacterial cell membrane by evaluating the leakage of intracellular K^+^ ion. They proposed that leakage of K^+^ from the cells reflects the antibacterial potency of the membrane disturbing compounds. The initial rate of leakage was considered as the damage to the cell membranes while the total amount of leaked K^+^ was evaluated as the antibacterial activity. Again, farnesol and nerolidol were the most effective compounds. Finally, they concluded that the length of the hydrocarbon chain connected to the hydroxyl group, plays an important role in antibacterial and cell membrane disrupting activity and it should be between C10 and C12 for appropriate effect against *S. aureus* [174]*.* Another terpene compound with antibacterial activity against *S. aureus* is dehydroabietic acid (54) which is a kind of resin acid [178]. It was found that dehydroabietic acid derivatives have antibacterial effects, too [179–181].

One of the most important terpenes that have the potential to be used in anti-infective therapy is carvone [173]. It has been shown while (4R)-(−)-carvone (55) was active against *Campylobacter jejuni*, *E. faecium* and *E. coli*, (4S)-(+)-carvone (56) was effective towards *L.monocytogenes* [182]. Both optical isomers were active towards various pathogenic fungi. In general, carvone inhibits the transformation of cellular yeast form of *C. albicans* to the filamentous form, that is responsible for the fungus pathogenicity [173].

The other antifungal compound is thymol (57) which has shown potent activity against clinical isolates of *C. albicans*, *Candida glabrata*, and *Candida krusei*, alone and in the combination of fluconazole. The MIC values of thymol were 49.37, 51.25 and 70 μg/ml for *C. albicans*, *C. glabrata* and *C. krusei* strains, respectively. Thymol also exhibited synergistic activity toward all tested species of *Candida* in combination with fluconazole [183]. Due to their potent and wide antifungal activity, Abbaszadeh et al. also suggested thymol and carvacrol (58) in addition to eugenol (59) and menthol (60) as good alternatives of synthetic fungicides in food industries. Indeed, they reported that all of these compounds were effective in different extents against various food-decaying fungi including *Aspergillus niger*, *Aspergillus fumigatus*, *Aspergillus flavus*, *Aspergillus ochraceus*, *Alternaria alternata*, *Botrytis cinerea*, *Cladosporium* spp., *Penicillium citrinum*, *Penicillium chrysogenum*, *Fusarium oxysporum*, and *Rhizopus oryzae* [184]. These observations were also confirmed by other research groups [185]. Althunibat et al. considered thymol as well as carvacrol as the major components of *Thymus capitatus*. The antibacterial effects of both compounds were evaluated against *E. coli*, *Enterobacter aerogenes*, *S. aureus*, and *P. aeruginosa*. In conclusion, the MIC values were 0.005–0.008 mg/mL for thymol and 0.007–0.008 mg/mL for carvacrol [186]. During biofilm formation of *Salmonella* spp. (*Salmonella typhimurium*, *Salmonella enteritidis* and *Salmonella saintpaul*), both substances reduced bacterial counts on polypropylene surface, about 1–2 log at subinhibitory concentrations; but for established biofilms about 1–5 log were observed at MIC or 2× MIC [187]. Although Chauhan et al. introduced “disruption of membrane integrity” as the main mechanism of thymol against *S. typhimurium* [188], Miladi et al hypothesize that the two major monoterpenic phenol, thymol, and carvacrol, acts through the inhibition of EP in a concentration-dependent manner. They found that these natural compounds enhanced the accumulation of ethidium bromide (EtBr) in foodborne pathogens through the inhibition of EtBr cell efflux [189].

Broniatowski et al. studied the antimicrobial mechanism of two pentacyclic triterpenes, ursolic acid (61) and α-amyrin (62) as the natural products with a broad spectrum of antibacterial activity. They used the Langmuir monolayer technique to model the interaction of the two compounds with the inner membrane of *E. coli*. Both pentacyclic triterpenes displayed disorganizing effects on the applied model of *E. coli* membrane [190].

The other famous terpenoids are eugenol and cinnamaldehyde (63) that are present in essential oils of several plants and demonstrated to be active against a wide spectrum of pathogens. After a comprehensive study on 30 strains of *H. pylori* as one of the major human pathogens involved in gastric and duodenal ulcers as well as gastric malignancy, Ali et al. revealed that the two bioactive compounds could prevent *H. pylori* growth without developing any resistance towards these compounds [191]. Eugenol also has exhibited notable bioactivity against biofilms of MRSA and MSSA clinical strains. According to the study of Yadav et al. eugenol inhibits biofilm construction, interrupts cell-to-cell communication, eradicates the pre-established biofilms, and kills the bacteria in biofilms, equally for MRSA and MSSA. These effects of eugenol were attributed to the impairment of the bacterial cell membrane and the leakage of the cell contents. Yadav et al. also reported that eugenol reduced the expression of genes related to the biofilm and enterotoxin production at a sub-inhibitory concentration [192]. In the study of Rathinam et al. eugenol exhibited comparable effects on biofilm formation and virulence factor synthesis of *P. aeruginosa* [193]. A study on the cinnamaldehyde mechanism of action against *E. coli* and *S. aureus* using scanning electron microscopy, showed that in the presence of cinnamaldehyde, the structure of bacterial membrane was damaged, membrane potential decreased and metabolic activity was affected which finally resulted in the bacterial growth inhibition [194].

Several terpenoid derivatives also exhibited antimycobacterial activity, as one of the most important pathogens. A list of such natural products with the emphasize on *M. tuberculosis* was reported by Copp, including sandaracopimaric acid, (+)-Totarol, Agelasine F, elisapterosin B, costunolide, parthenolide, 1,10-epoxycostunolide, santamarine, reynosin, alantolactone, puupehenone, elatol, deschloroelatol, debromolaurinterol, allolaurinterol, and aureol. This antimycobacterial activity is related to the usually moderate to the high lipophilic structure of terpene derivatives which facilitate their penetration into the mycobacterial cell wall [195].

## Conclusions and perspectives

There are lots of evidence suggesting that medicinal plants are very effective in the treatment of infectious diseases. The plants hold great promise as a source of novel antimicrobial agents. They are readily available, cheap and also, almost; do not have any side effects. Plant derivative compounds including phytochemicals have even been employed to treat various infectious disease and have shown interesting antimicrobial activity against several human pathogens. Some of these compounds show both intrinsic antibacterial activity and antibiotic resistance modifying activities. Some of them while not effective by themselves as antibiotics, when combined with antibiotics, they can overcome antibiotic resistance in bacteria. Co-administration of them with antibiotics leads to reduce the MIC values of antibiotics and synergistic effects were observed.

Similar to co-administration of antibiotics with a different mechanism of actions such as amoxicillin-clavulanate and isoniazid-rifampicin-pyrazinamide-ethambutol, the combination of conventional antibiotics and natural compounds which act on the different target sites of bacteria leads to superior efficacy and can be quite successful, especially in suppressing the development of resistance. Hence, it is necessary to understand the exact molecular mechanism of the compound. Furthermore, getting to know antimicrobial plant mechanisms may be useful in developing a novel therapeutic approach. With this regard, various mechanisms have been suggested to explain the mode of action and generally include damaging the bacterial cell membrane, inhibiting EPs and also inhibiting DNA and protein biosynthesis. For instance, a combination of EGCG and tetracycline, a protein synthesis inhibitor antibiotic, led to synergistic effects. EGCG inhibited the efflux pump of bacteria and therefore the intracellular concentration of tetracycline was increased and consequently, could have a direct impact on restoring antibiotic efficacy in resistant strains.

In conclusion, in recent years, people have been paying more attention to herbal-based medicines due to their properties. However, many studies still need to be conducted to ensure the mechanism of action and also the safety of antimicrobial phytochemicals.

### Challenges and future perspective

The translation of in vitro studies to in vivo experiments and finally to human clinical trials has been the major challenge in the development of new phytochemicals. In the case of natural antimicrobial agent, the problem is more serious since many factors can affect the efficacy of the natural product including tissue penetration, maximum plasma concentration can be achieved and bioavailability. As an instance, phenolic natural products are readily glucuronidated with hepatic enzymes that dramatically affects their tissue penetration and maximum plasma concentration.

The spread of antibiotic-resistant microorganisms has been a big threat to successful therapy of microbial infections. So far, there is an urgent need to develop new strategy to combat antibiotic resistance. Phytochemicals which are characterized by diverse chemical structures and mechanisms of action are attractive therapeutic tools for discovering bioactive products in the next years. However, continued researches should be carried out for a better understanding of the exact mechanisms and also pharmacodynamic and pharmacokinetic properties of the molecules.

## Data Availability

This review was based on data extracted from published papers available in all relevant databases without limitation up to 15th December 2018.

## Abbreviations

ABC: ATP binding cassette
EGCG: Epigallocatechin gallate
EP: Efflux pump
MATE: Multidrug and toxic efflux
MDR: Multidrug-resistant
MFS: Major facilitator superfamily
RND: Resistance-nodulation-division
SMR: Small multidrug resistance
